# Clinical results and failure rates after meniscal allograft transplantation and autologous chondrocyte implantation: a systematic review

**DOI:** 10.1186/s43019-025-00291-4

**Published:** 2025-09-22

**Authors:** Johannes Pawelczyk, Ilias Fanourgiakis, Sven Feil, Sven Schneider, Ioannis Kougioumtzis, Rainer Siebold

**Affiliations:** 1 International Center for Orthopedics, ATOS Clinic Heidelberg, Bismarckstr. 9-15, 69115 Heidelberg, Germany; 2 Medical Faculty Heidelberg, Heidelberg University, Heidelberg, Germany; 3 Center for Preventive Medicine and Digital Health, Medical Faculty Mannheim, Heidelberg University, Mannheim, Germany; 4 Institute for Anatomy and Cell Biology, Heidelberg University, Heidelberg, Germany

**Keywords:** Meniscal allograft transplantation, Autologous chondrocyte implantation, Osteoarthritis, Meniscus, Cartilage

## Abstract

**Background:**

Meniscal allograft transplantation (MAT) and autologous chondrocyte implantation (ACI) are well-established procedures. However, evidence regarding the safety and efficacy of their combined application remains inconclusive. Thus, the present systematic review aimed to comprehensively evaluate the existing literature on clinical outcomes after combined MAT and ACI.

**Methods:**

A comprehensive search of clinical studies reporting on clinical outcomes after combined MAT and ACI was performed across three databases in accordance with the review protocol. Key demographic data, surgical technique, knee-specific patient-reported outcome measures, failure rates, and reoperation rates were extracted from eligible studies and analyzed. Risk of bias was assessed using the Methodological Index for Non-Randomized Studies (MINORS).

**Results:**

The search yielded 246 studies, 9 of which satisfied inclusion and exclusion criteria, comprising 155 patients undergoing combined MAT and ACI at an average age of 36.1 years. The mean follow-up duration was 5.3 years (2.0–12.9 years). Clinical outcome measures improved across all studies (with one exception in a single outcome measure); statistical significance was shown in six out of seven studies reporting significance analysis. International Knee Documentation Committee (IKDC) scores were reported by four studies, showing an average improvement of 22.0 points. Lysholm scores showed an average improvement of 20.0 points across five studies. Of five studies comparing combined procedures with isolated procedures, one comparative study and one literature comparison reported inferior outcomes after combined surgery, while one subgroup analysis and two literature comparisons found comparable outcomes. Failure rates ranged from 0.0% to 52.6%, with significant methodological heterogeneity. The mean reoperation rate was 35.0% across seven studies.

**Conclusions:**

Combined MAT and ACI leads to meaningful improvements in clinical outcomes, with notable failure and reoperation rates. While some studies suggest outcomes may be comparable or inferior to isolated procedures, only one study directly assessed this. As such, definitive conclusions cannot be drawn. Combined MAT and ACI can be considered as a viable option in select patients, but realistic expectations must be ensured.

**Supplementary Information:**

The online version contains supplementary material available at 10.1186/s43019-025-00291-4.

## Background

With an estimated prevalence of 222 per 100,000, meniscal tears are a frequent pathology in orthopedic sports medicine [1]. Significant progress has been made regarding meniscal preservation, resulting in a decreasing incidence of meniscectomies [2]. However, meniscectomy is still a relatively common procedure, leading to accelerated joint degeneration and osteoarthritis progression [3–5].

Meniscal allograft transplantation (MAT) has emerged as a viable surgical approach to restore meniscal function and delay degenerative changes, with substantial evidence supporting its safety and efficacy [6–9]. Similarly, autologous chondrocyte implantation (ACI) has become one of the foremost regenerative procedures for the surgical treatment of focal chondral defects [10–12]. In patients with meniscal insufficiency and significant coexisting chondral damage, combined MAT and cartilage restoration may offer improved outcomes by simultaneously restoring meniscal and chondral integrity, and some studies have started to advocate multipronged surgical approaches [7, 13–15].

Despite the theoretical advantages of combined MAT and ACI, existing literature on clinical outcomes, graft survival, and reoperation rates is limited. While numerous studies have addressed isolated MAT [9] or ACI [10, 11], few have investigated the implications of combining these specific procedures. It remains unclear whether combined MAT and ACI can significantly improve key outcome measures at an acceptable risk level, or conversely, whether they predispose patients to higher failure and reoperation rates and inferior clinical outcome improvements.

Thus, the purpose of this systematic review was to address this knowledge gap by comprehensively evaluating the existing evidence on clinical outcomes after combined MAT and ACI, to assess the viability, safety, and efficacy of this combined approach. The primary hypothesis was that combined MAT and ACI would achieve meaningful improvements in clinical outcome measures, comparable to those after each procedure performed in isolation, at acceptable failure and reoperation rates.

## Methods

### Standard protocol approvals, registration, and informed consent

The protocol of this systematic review has been prospectively registered in the International Prospective Register of Ongoing Systematic Reviews (PROSPERO, ID: CRD42022349829) and published separately. This review is presented in compliance with the updated Preferred Reporting Items for Systematic Reviews and Meta-Analyses (PRISMA) [16]. Institutional review board approval and written informed consent were not required, according to the study design.

### Data sources, searches, and study selection

A comprehensive systematic literature search was performed to identify eligible studies reporting clinical outcomes after combined MAT and ACI. The search was performed separately by two independent reviewers (J.P., I.F.), querying the following three databases: (i) Medline via PubMed and PubMed Central, (ii) Web of Science Core Collection, and (iii) Cochrane Library. Search strings included “meniscal allograft transplantation” and “autologous chondrocyte implantation”. The complete search strategy is detailed in the supplementary materials [see Additional file 1].

### Eligibility criteria

All articles matching the search terms were manually assessed and discussed among the reviewers. A decision regarding inclusion was made on the basis of the eligibility criteria set forth in the systematic review protocol (Table 1). The first and second author (J.P., I.F.) screened all 246 records independently, with both reviewers being blinded. While no formal interrater reliability metrics were calculated, high interrater agreement was observed, and any disagreements were resolved by mutual consensus after discussion with the senior author.

**Table 1:** Eligibility criteria

Inclusion criteria	Exclusion criteria
Studies reporting clinical outcomes after *combined* MAT and ACI	Studies reporting results after *isolated* MAT or ACI
Studies addressing meniscus-deficient knees with concomitant high-grade chondral lesions	Studies reporting results after meniscal replacement with synthetic meniscus implants
Minimum follow-up period of 12 months	Studies classified as case reports, narrative or systematic reviews, commentaries, editorials, consensus statements, or preprints, or follow-up period < 12 months
Level I, II, III, or IV evidence according to the Oxford Centre for Evidence Based Medicine	Studies with less than five patients receiving combined MAT and ACI
English language	
Human subjects	
Year of publication between 1992 and 2024	

Eligibility criteria as defined in the systematic review protocol (published separately)

*MAT* meniscal allograft transplantation, *ACI* autologous chondrocyte implantation

The search was updated toward the end of the systematic review to ensure comprehensiveness of the bibliography, including any recently published studies. All data generated or analyzed during this study are included in this article and its supplementary material.

### Selection criteria for combined MAT and ACI

Combined MAT and ACI is typically reserved for symptomatic patients under 50 years of age with prior subtotal or total meniscectomy and focal, contained chondral lesions (ICRS grade III–IV, typically 2–10 cm^2^) who remain symptomatic despite conservative management for at least 6 months. Ideal candidates have normal or correctable limb alignment, stable knee ligaments, and limited articular cartilage damage amenable to repair. Contraindications include advanced osteoarthritis (Kellgren–Lawrence grade > 2), uncorrectable malalignment or instability, diffuse chondral damage, active infection, or inflammatory arthropathy [7, 17].

### Outcomes measures

Extracted clinical outcome measures were qualitatively assessed and compared. The primary outcome measure of interest was the International Knee Documentation Committee (IKDC) subjective score, as it has been shown to have acceptable psychometric performance for the outcomes of both meniscus and cartilage injuries of the knee [18, 19]. Secondary outcome measures comprised: (i) other clinical outcome measures reported in the included studies, (ii) failure rates, (iii) reoperation rates, (iv) complications, and (v) subsequent surgeries.

### Bias assessment and data extraction

Risk of bias was assessed using the Methodological Index for Non-Randomized Studies (MINORS) [20], by two independent, blinded reviewers (J.P., I.F.). Disagreements were resolved by consensus after discussion with the senior author. Data from the following categories were manually extracted for each eligible study: date of publication, study design, patient/defect demographic data, surgical techniques, clinical outcome measures, failure rates, reoperation rates, and complications.

Due to the small number of included studies, significant methodological heterogeneity, and absence of a common effect measure across all studies, a formal funnel plot analysis for publication bias was not feasible. The heterogeneity in outcome measures, follow-up periods, and surgical techniques precluded meaningful visual assessment of publication bias through this method.

### Statistical analysis

A limited explorative analysis was performed as available data permitted to assess outcomes of interest among eligible articles. Descriptive statistics were used to present the main characteristics of studies included in the review. Due to significant methodological heterogeneity, no meta-analysis was performed. Arithmetic mean and standard deviation (SD) were calculated for relevant outcome measures. Continuous outcomes were assessed by raw means. Categorical variables were presented as counts and percentages. Continuous variables are reported as mean ± SD, unless otherwise stated. All statistical analyses were performed with Microsoft Excel for Mac version 16.92.

## Results

**Fig. 1:**
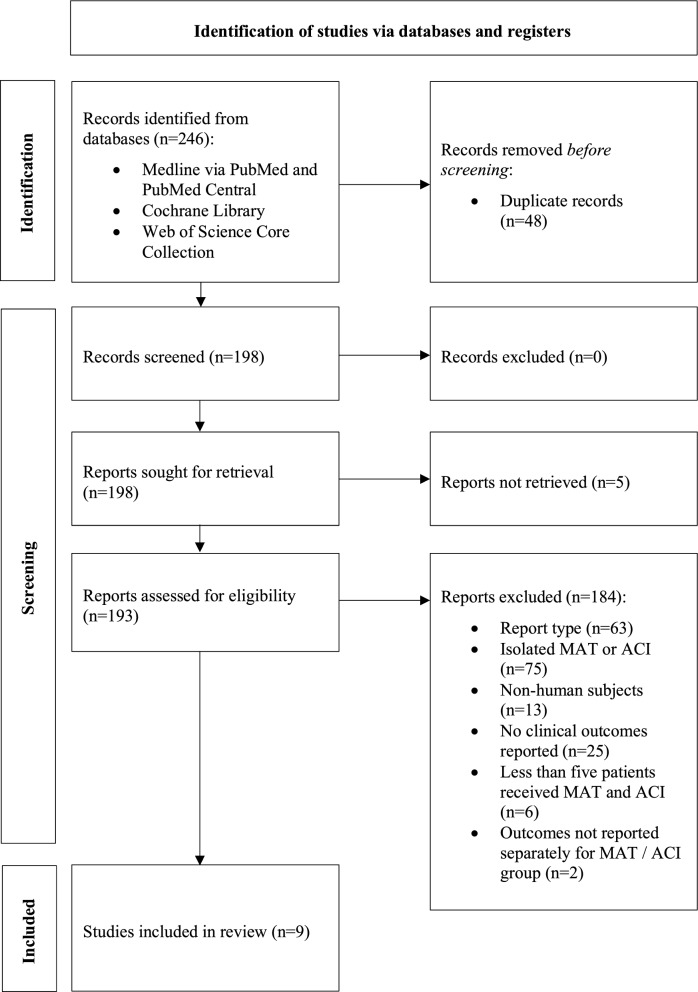
Screening and selection. Preferred Reporting Items for Systematic reviews and Meta-Analyses (PRISMA) flow chart. *MAT* meniscal allograft transplantation, *ACI* autologous chondrocyte implantation

The search yielded 246 studies, 9 of which satisfied inclusion and exclusion criteria [21–29], comprising 155 patients undergoing combined MAT and ACI at an average age of 36.1 years and average body mass index (BMI) of 25.7 kg/m^2^ (see Fig. 1, Table 2). The mean follow-up duration was 5.3 years (2.0–12.9 years). The mean chondral lesion size was 6.6 cm^2^. Patient and defect demographic data are detailed in Table 3. Surgical and graft preservation techniques were comparatively heterogeneous and are detailed in Table 4.

**Table 2:** Included studies

Study	Year	Journal	DOI
Álvarez-Lozano	2022	*Musculoskelet Surg*	10.1007/s12306-020-00680-w
Yoon	2019	*Arch Orthop Trauma Surg*	10.1007/s00402-019-03148-0
Pawelczyk	2024	*Knee Surg Sports Traumat Arthrosc*	10.1002/ksa.12023
Ogura	2016	*Orthop J Sports Med*	10.1177/2325967116668490
Bhosale	2007	*Knee*	10.1016/j.knee.2007.07.002
Farr	2007	*Am J Sports Med*	10.1177/0363546507301257
Cvetanovich	2017	*Am J Sports Med*	10.1177/0363546516663711
Rue	2008	*Am J Sports Med*	10.1177/0363546508317122
Gersoff	2002	*Oper Tech Sports Med*	n.a.

Comprehensive list of all studies included in the present systematic review

*DOI* digital object identifier, *n.a.* not applicable

**Table 3:** Patient and defect demographic data

Study	Number of patients	Male	Female	Average age, years	Medial MAT	Lateral MAT	Defect size, cm^2^	Average FU, months
Álvarez-Lozano	16	9	7	56.0 ± 6.0	n.r.	n.r.	n.r.	24.0
Yoon	19	16	3	34.8 ± 8.4	8	11	4.2 ± 1.8	154.8 ± 25.2
Pawelczyk	20	13	7	31.9 ± 9.7	5	15	4.6 ± 3.6	72.6 ± 34.4
Ogura	17	9	8	31.7 ± 10.8	1	17	7.6 ± 5.3	94.8 ± 58.8
Bhosale	8	7	1	42.5 ± 10.1	2	6	10.74 ± 5.6	38.28 ± 18.0
Farr	29	23	6	36.9 ± 9.1	21	8	n.r.	54 ± 15.6
Cvetanovich	12	n.r.	n.r.	n.r.	2	10	n.r.	n.r.
Rue	15	5	11	23.4	n.r.	n.r.	3.9	40.8
Gersoff	19	11	8	31.2	11	8	8.4	24.72

Values are reported as counts or arithmetic mean ± standard deviation (SD) or counts

*n.r.* not reported, *MAT* meniscal allograft transplantation, *FU* follow-up

**Table 4:** Surgical techniques

Study	MAT graft type	MAT fixation technique	MAT preservation technique	ACI generation	Surgical approach
Álvarez-Lozano	Meniscus + full tibial plateau	Screws/plates	Fresh	Third	Open
Yoon	n.r.	Bone-plug/keyhole	Fresh-frozen	First	Open
Pawelczyk	Bone-bridge	Bridge-in-slot	Fresh-frozen	n.r.	Arthroscopic/miniarthrotomy
Ogura	Bone-block	Keyhole/bridge-in-slot	Fresh-frozen/cryopreserved	First/second	Open
Bhosale	Bone-plug	Transtibial sutures	Cryopreserved	First/second	Open
Farr	Bone-bridge	Slot, transtibial sutures/interference screw	Fresh-frozen	First	Miniarthrotomy
Cvetanovich	n.r.	n.r.	n.r.	First/second	n.r.
Rue	Bone-bridge/bone-plug	Bone-plug/keyhole, bridge-in-slot	Cryopreserved/fresh-frozen	First	n.r.
Gersoff	Bone-plug/keyhole/trough	Press-fit, interference screw, suture anchors	n.r.	First	Open

Detailed description of surgical techniques used by each author for MAT and ACI, respectively

*MAT* meniscal allograft transplantation, *ACI* autologous chondrocyte implantation, *MACI* matrix-associated autologous chondrocyte implantation, *n.r.* not reported

Clinical outcome measures improved across all studies (except for Rue et al. [27], where average 12-Item Short Form Survey (SF-12) mental scores deteriorated by 3.5 points). Improvements in clinical outcome measures were statistically significant in six out of seven studies reporting significance analysis. Improvements were most consistent in KOOS Sports and Quality of Life subdomains, with mixed results in Western Ontario and McMaster Universities Arthritis Index (WOMAC) pain and function scores. Reported outcome measures varied considerably between studies. IKDC scores showed an average improvement of 22.0 points across four studies [22, 26–28]. Lysholm scores showed an average improvement of 20.0 points across five studies [22, 24, 25, 27, 28]. Full clinical outcome data are detailed in Table 5 and Table 6.

**Table 5:** Difference between pre- and postoperative outcome scores

Study	IKDC	Lysholm	Tegner	KSSR	VAS pain	VAS satisfaction	KL	Noyes score		Cincinnati score		
								Sports	Symptoms	Patient	Modified	Normal
Álvarez-Lozano	n.r.	n.r.	n.r.	+60.6	n.r.	n.r.	n.r.	n.r.	n.r.	n.r.	n.r.	n.r.
Yoon	** +10.2**	** +12.9**	** +1.5**	n.r.	n.r.	n.r.	n.r.	n.r.	n.r.	n.r.	n.r.	n.r.
Pawelczyk	** +16.4**	** +17.0**	** +0.5**	n.r.	n.r.	+1.8	n.r.	n.r.	n.r.	n.r.	n.r.	n.r.
Ogura	n.r.	n.r.	n.r.	n.r.	**−4.0**	n.r.	+0.2	n.r.	n.r.	n.r.	** +3.7**	n.r.
Bhosale	n.r.	17.0	n.r.	n.r.	n.r.	n.r.	n.r.	n.r.	n.r.	n.r.	n.r.	n.r.
Farr	n.r.	** +28.5**	n.r.	n.r.	**−2.5**	n.r.	n.r.	n.r.	n.r.	** +2.4**	** +2.3**	n.r.
Cvetanovich	** +30.9**	n.r.	n.r.	n.r.	n.r.	n.r.	n.r.	n.r.	n.r.	n.r.	n.r.	n.r.
Rue	** +30.5**	** +24.4**	** +1.8**	n.r.	n.r.	n.r.	n.r.	** +19.3**	** +2.4**	n.r.	n.r.	n.r.
Gersoff	n.r.	n.r.	n.r.	n.r.	n.r.	n.r.	n.r.	n.r.	n.r.	n.r.	n.r.	+4.9

Difference between mean pre- and postoperative outcome measures. Statistically significant differences are highlighted in bold

*IKDC* International Knee Documentation Committee, *KSSR* Knee Society Score Rating, *VAS* visual analogue scale, *KL* Kellgren–Lawrence score, *KOOS* Knee Injury and Osteoarthritis Outcome Score, *WOMAC* Western Ontario and McMaster Universities Arthritis Index, *SF-36* 36-Item Short Form Survey, *SF-12* 12-Item Short Form Survey, *ADL* activities of daily living, *QoL* quality of life, *PCS* physical component summary, *MCS* mental component summary, *n.r.* not reported

**Table 6:** Difference between pre- and postoperative outcome scores (continued)

Study	KOOS					WOMAC				SF-36		SF-12	
	Pain	Symptoms	ADL	Sport	QoL	Total	Pain	Stiffness	Function	PCS	MCS	PCS	MCS
Álvarez-Lozano	+5.14					n.r.	n.r.	n.r.	n.r.	n.r.	n.r.	n.r.	n.r.
Pawelczyk	** +23.2**	+16.1	** +18.8**	** +23.3**	** +22.5**	n.r.	n.r.	n.r.	n.r.	n.r.	n.r.	n.r.	n.r.
Ogura	n.r.	n.r.	n.r.	n.r.	n.r.	**−26.5**	**−6.5**	**−1.7**	**−18.2**	** +10.6**	** +6.7**	n.r.	n.r.
Cvetanovich	n.r.	n.r.	n.r.	n.r.	** +34.6**	n.r.	n.r.	n.r.	n.r.	n.r.	n.r.	n.r.	n.r.
Rue	** +26.0**	** +16.9**	** +14.8**	** +40.8**	** +32.2**	n.r.	n.r.	n.r.	n.r.	n.r.	n.r.	** +5.0**	−3.5

Difference between mean pre- and postoperative outcome measures. Improvements were most consistent in KOOS Sports and Quality of Life subdomains, with mixed results in WOMAC pain and function scores. Statistically significant differences are highlighted in bold

*IKDC* International Knee Documentation Committee, *KSSR* Knee Society Score Rating, *VAS* visual analogue scale, *KL* Kellgren–Lawrence score, *KOOS* Knee Injury and Osteoarthritis Outcome Score, *WOMAC* Western Ontario and McMaster Universities Arthritis Index, *SF-36* 36-Item Short Form Survey, *SF-12* 12-Item Short Form Survey, *ADL* activities of daily living, *QoL* quality of life, *PCS* physical component summary, *MCS* mental component summary, *n.r.* not reported

Of five studies [22, 24–27] comparing combined MAT and ACI with these procedures performed in isolation, one comparative study (*n* = 33) [22] and one literature comparison [25] reported inferior outcomes after combined surgery, while one subgroup analysis (*n* = 37) [26] and two literature comparisons [24, 27] found comparable outcomes. Failure rates ranged from 0.0% to 52.6% across seven studies [21–23, 25, 27–29]. However, the definition of “failure” varied widely or was omitted entirely. The mean reoperation rate was 35.0% across seven studies [22, 23, 25–29]. Data regarding failures and reoperations are detailed in Table 7.

**Table 7:** Failure and reoperation rates

First author	Failure rate, %	Definition of failure	Reoperation rate, %
Álvarez-Lozano	18.8	Conversion to arthroplasty	n.r.
Yoon	52.6	ACI: need for revision surgery due to unrelieved symptoms, ICRS IV° cartilage defect (MRI), or postoperative Lysholm score < 65; failure rate calculated on the basis of number of patients with ACI failure at final follow-up	5.3
Pawelczyk	15.0	Conversion to arthroplasty or revision MAT/ACI/similar cartilage procedure	40.0
Ogura	35.3	Conversion to arthroplasty, revision cartilage repair/MAT, new defects necessitating additional surgery	58.0
Bhosale	n.r.	n.r.	n.r.
Farr	13.8	Revision MAT/ACI, conversion to arthroplasty	69.0
Cvetanovich	n.r.	n.r.	25.0
Rue	0.0	MAT/cartilage repair revision or arthroscopically confirmed MAT/cartilage failure	26.7
Gersoff	5.3	n.r.	21.1

Failure rates, definitions of failure events, and reoperation rates, as reported in each study

*MAT* meniscal allograft transplantation, *ACI* autologous chondrocyte implantation, *n.r.* not reported

Risk of bias was assessed using the MINORS instrument [20]. The results are presented in Table 8. One comparative [22] and eight noncomparative studies [21, 23–29] were analyzed. The average percentage of the ideal score achieved was 67%, indicating moderate quality.

**Table 8:** Risk of bias assessment

Study	Clearly stated aim	Consecutive patients	Prospective data collection	Appropriate endpoints	Unbiased assessment of endpoints	Appropriate FU	Loss to FU < 5%	Prospective calculation of study size	Adequate control group	Contemporary groups	Baseline equivalence of groups	Adequate statistical analysis	% of ideal score
Álvarez-Lozano	2	2	2	2	0	2	2	0	n.a.	n.a.	n.a.	n.a.	75%
Yoon	2	2	1	2	2	2	0	2	2	2	2	2	88%
Pawelczyk	2	2	2	2	2	2	1	1	n.a.	n.a.	n.a.	n.a.	88%
Ogura	1	2	2	2	0	2	1	0	n.a.	n.a.	n.a.	n.a.	63%
Bhosale	1	2	1	2	1	2	2	0	n.a.	n.a.	n.a.	n.a.	69%
Farr	2	2	2	2	0	2	1	0	n.a.	n.a.	n.a.	n.a.	69%
Cvetanovich	2	1	2	2	0	2	1	0	n.a.	n.a.	n.a.	n.a.	63%
Rue	2	2	2	2	0	2	1	0	n.a.	n.a.	n.a.	n.a.	69%
Gersoff	0	0	0	1	0	2	0	0	n.a.	n.a.	n.a.	n.a.	19%

Risk of bias assessment using the Methodological Index for Non-Randomized Studies (MINORS); 0, not reported; 1 reported but inadequate; and 2, not reported. Columns 10–13 only apply to comparative studies. The maximum score for noncomparative and comparative studies is 16 and 24, respectively

*FU* follow-up, *n.a.* not applicable

Specific indications for performing combined MAT and ACI were explicitly reported in only four of nine studies. Yoon et al. [22] included patients < 50 years with normal alignment (< 3° varus/valgus) and ICRS grade IV lesions ≥ 1 cm. Ogura et al. [23] required full-thickness defects with meniscal deficiency resistant to nonoperative therapy. Cvetanovich et al. [26] indicated surgery for symptomatic grade IV lesions, excluding patients with osteoarthritic changes or poor rehabilitation compliance. Gersoff et al. [29] provided the most comprehensive criteria, recommending combined procedures for patients aged 50–55 years without systemic disease and with isolated cartilage damage and meniscal deficiency, emphasizing the need to address any instability or malalignment.

## Discussion

The main findings of the present review are: (i) combined MAT and ACI consistently achieves meaningful improvements in established outcome measures at notable failure and reoperation rates, (ii) results after combined MAT and ACI are inferior or comparable to those after each procedure performed in isolation, but specific evidence is limited, and (iii) overall evidence on combined MAT and ACI is scarce and heterogeneous, warranting further investigation and standardization.

### Clinical outcomes

All studies reported improvements across all clinical outcome measures at final follow-up after combined MAT and ACI compared with findings before surgery, except for a single study and outcome measure [27]. Mean improvements in key established outcome metrics (e.g., IKDC, Lysholm, and KOOS) exemplify relevant and meaningful improvements in function and symptoms. As such, the average improvement in IKDC scores across four studies reached 22.0 points, vastly exceeding the minimal clinically important difference (MCID) for MAT of 9.9 points, as defined by Liu et al. [30], and the average improvement in Lysholm scores across five studies reached 20 points, likewise exceeding the corresponding MCID of 12.3 points [30]. This supports combined MAT and ACI as an effective treatment approach, able to consistently achieve significant and relevant improvements in established outcome scores.

Comparisons between the results of combined surgery and those after each procedure performed in isolation were less clear. Farr et al. [31] reported that their results after combined MAT and ACI were inferior to literature-reported results after each procedure performed separately. However, they did not clearly state what data they compared against. To the contrary, Rue et al. [27], who compared combined MAT and ACI with combined MAT and osteochondral allograft (OA), reported that results after combined MAT and ACI were comparable to published reports of these procedures performed in isolation. The comparison between ACI and OA as a concomitant procedure was largely inconclusive, with superior absolute IKDC and KOOS pain/symptoms/activities of daily living (ADL)/sports/quality of life (QoL) scores for ACI, but limited comparability of the groups, due to significant differences in age, defect size, and preoperative scores. As a combined cohort, they report significant improvements across all outcome measures, except SF-12 mental.

Yoon et al. [22], the only study directly and specifically comparing ACI and combined MAT and ACI, concluded that combined surgery did not improve clinical outcomes as much as isolated ACI, but patients who had received isolated ACI were younger on average and had smaller chondral defects. Cvetanovich et al. [26] found no adverse effect of concurrent MAT on ACI performed in adolescent patients and Bhosale et al. [24] also reported no negative effects of combining these techniques. Ultimately, the limited existing literature is rather inconclusive regarding possibly inferior outcome improvements when combining MAT and ACI, compared with isolated MAT or ACI.

On the basis of this systematic analysis of the limited available literature, clinical improvements after combined MAT and ACI are expected to be slightly inferior or comparable to those after each procedure performed in isolation, while consistently yielding meaningful improvements in established outcome scores compared with preoperative values.

### Failure and reoperation rates

Reported failure rates ranged from 0.0% to 52.6%, with a high level of heterogeneity in the operationalization of “failure.” Álvarez-Lozano et al. [21] defined failure simply as conversion to total knee arthroplasty. Accordingly, they observed a comparatively low failure rate of 18.8%. On the contrary, Rue et al. [27] defined failure as either MAT revision, cartilage repair revision, or arthroscopically confirmed MAT or cartilage failure, but reported a failure rate of 0.0%. Reoperation rates varied significantly, with an average of 35.0%. Arguably, these overall failure and reoperation rates are notable, but acceptable, considering the lack of alternative treatment approaches. However, it is critical to inform patients about a significant risk of failure or subsequent surgery to ensure realistic expectations.

The observed failure and reoperation rates must be interpreted within the context of isolated MAT outcomes. Systematic reviews report MAT survivorship of only 73.5% at 10 years and 60.3% at 15 years [9], indicating substantial baseline failure rates even without concurrent procedures. Patients requiring combined MAT and ACI likely represent more complex cases with greater chondral damage and worse overall joint pathology compared with those undergoing isolated MAT, potentially explaining the comparable or slightly higher failure rates observed. The mean reoperation rate of 35.0% in the present review, while notable, aligns with the 32% reoperation rate reported for isolated MAT [17]. Thus, the failure rates observed with combined procedures may primarily reflect patient selection factors and the inherent challenges of meniscal transplantation rather than specific complications from performing simultaneous procedures.

### Heterogeneity in failure definitions

The dramatic variation in reported failure rates (0.0-52.6%) is likely attributable primarily to inconsistent failure definitions across studies (see Table 7). As such, some studies defined failure as requiring any subsequent surgery, while others considered only conversion to arthroplasty. This significantly limits the comparability of reported failure rates. For instance, Yoon et al.’s comprehensive definition—including revision surgery, ICRS grade IV defects on magnetic resonance imaging (MRI), or Lysholm scores < 65—yielded a 52.6% failure rate, while Álvarez-Lozano et al.’s narrow definition of conversion to arthroplasty alone resulted in 18.8%. Most striking is Rue et al.’s 0.0% failure rate using arthroscopic confirmation criteria, despite documenting a 26.7% reoperation rate, illustrating how restrictive definitions can mask treatment challenges. This heterogeneity represents a significant issue, limiting comparability and generalizability of the findings in the present review. Without standardized outcome reporting, the true failure rate of combined MAT and ACI remains unknowable, and clinicians must interpret these widely varying rates with extreme caution.

### Analysis of failure causes

Deep analysis of failure causes was severely limited by the available data. Several studies reported only failure rates without detailing specific failure modes, surgical findings at reoperation, or distinguishing between meniscal versus cartilage failures. No studies provided systematic categorization of whether failures were primarily meniscal, ACI-related, or due to progressive joint deterioration. This represents a fundamental limitation of the current literature that prevents evidence-based understanding of why these combined procedures fail and precludes the development of strategies to improve outcomes.

### Data availability, quality of evidence, and risk of bias

Only nine studies matching the eligibility criteria were found. All studies had small sample sizes and a low level of evidence [32]. Furthermore, the risk of bias assessment using the MINORS instrument indicated only moderate quality. Significant heterogeneity in methodology and reported outcome measures was observed. Most, but not all, studies reported surgical techniques for both MAT and ACI in detail and disclosed meniscal allograft preservation techniques (i.e., fresh-frozen), as well as overall surgical approach (i.e., arthrotomy). Conversely, studies in which only a subgroup of patients received combined MAT and ACI often did not report pertinent clinical outcome measures and patient demographic data separately. This highlights a need for further investigation and standardization of outcome reporting to enable better data aggregation, enabling robust comparisons for this specialized procedure.

### Publication bias and quality of evidence

The overall quality of evidence in this systematic review is low, with no randomized controlled trials identified and all included studies representing level III or IV evidence. The retrospective nature of most included studies (seven of nine) and the lack of control groups introduces substantial risk of bias and may lead to overestimation of treatment effects. Publication bias is a particular concern in this field, as studies reporting positive outcomes are more likely to be published than those showing neutral or negative results. The absence of prospectively registered trials and the lack of negative outcome reporting in the included studies further compounds this issue. Additionally, the small sample sizes (mean 17.2 patients per study) and single-center designs limit generalizability. These factors collectively suggest that the reported improvements in clinical outcomes, while encouraging, should be interpreted with caution. The true effect size of combined MAT and ACI may be smaller than reported in the current literature, and the high reoperation rates may actually represent an underestimate given the potential for selective reporting. Future research should prioritize prospective, controlled studies with preregistered protocols to provide more robust evidence for clinical decision-making.

### Mental health considerations

Notably, Rue et al. [27] reported a decline in SF-12 mental component scores (−3.5 points) despite improvements in physical outcomes, representing the only negative outcome change across all studies included in the present systematic review. This unexpected finding may reflect several factors: the psychological burden of extensive rehabilitation following combined procedures, unmet expectations regarding return to preinjury activity levels, or the emotional impact of high reoperation rates. The prolonged recovery from simultaneous procedures may contribute to psychological distress, particularly in younger, active patients accustomed to higher activity levels. However, as this finding was isolated to one study and other studies did not systematically assess mental health outcomes, its significance remains unclear, and it may simply reflect statistical noise. Future studies should routinely include mental health measures to better understand the psychological impact of these complex procedures.

### Limitations

This review has several notable limitations warranting discussion. First, the number of included studies and patients was low, due to the scarcity of existing evidence. Furthermore, very few studies directly compared combined MAT and ACI with isolated procedures, and the level of evidence of included studies was low. This is compounded by significant heterogeneity in surgical techniques, reporting, and research methodology, which precluded the performance of a meta-analysis. Failure rates were particularly difficult to compare, as definitions varied widely or were omitted entirely. Surgical techniques for MAT and ACI varied significantly. MAT preservation and fixation techniques were highly heterogeneous, ranging from meniscal allografts with full lateral or medial tibial plateaus to bone-plug or keyhole techniques. Finally, formal, quantitative interrater reliability statistics were not calculated during the screening process, which may affect the reproducibility of our study selection, though disagreements between reviewers were infrequent and resolved through mutual consensus after discussion with the senior author.

## Conclusions

Combined MAT and ACI leads to meaningful improvements in clinical outcomes, with notable failure and reoperation rates. While some studies suggest outcomes may be comparable or inferior to isolated procedures, only one study directly assessed this. As such, definitive conclusions cannot be drawn. Combined MAT and ACI can be considered as a viable option in select patients, but realistic expectations must be ensured.

## Supplementary Information

Supplementary material 1. Table 1 Exact search string used for the comprehensive literature search in each database.

## Data Availability

The datasets used and/or analyzed during the current study are available from the corresponding author on reasonable request.

## Abbreviations

MAT: Meniscal allograft transplantation
ACI: Autologous chondrocyte implantation
IKDC: International Knee Documentation Committee
PROSPERO: Prospective Register of Ongoing Systematic Reviews
PRISMA: Preferred Reporting Items for Systematic Reviews and Meta-Analyses
MINORS: Methodological Index for Non-Randomized Studies
SD: Standard deviation
BMI: Body mass index
DOI: Digital object identifier
n.a.: Not applicable
n.r.: Not reported
FU: Follow-up
MACI: Matrix-associated autologous chondrocyte implantation
KOOS: Knee Injury and Osteoarthritis Outcome Score
MCID: Minimal clinically important difference
OA: Osteochondral allograft
ADL: Activities of daily living
QoL: Quality of life

## References

[1] Ahmed I, Radhakrishnan A, Khatri C, Staniszewska S, Hutchinson C, Parsons N et al. (2021) Meniscal tears are more common than previously identified, however, less than a quarter of people with a tear undergo arthroscopy. Knee Surg Sports Traumatol Arthrosc 29(11):3892–3898. 10.1007/s00167-021-06458-2 33521890 10.1007/s00167-021-06458-2 PMC8514344

[2] Bergstein VE, Ahiarakwe U, Haft M, Mikula JD, Best MJ (2024) Decreasing incidence of partial meniscectomy and increasing incidence of meniscus preservation surgery from 2010 to 2020 in the United States. Arthroscopy. 10.1016/j.arthro.2024.07.030 39128681 10.1016/j.arthro.2024.07.030

[3] Espejo-Reina A, Sevillano-Pérez E, Espejo-Reina MJ, Lombardo-Torre M, Pérez-Blanca A, Espejo-Baena A (2023) The proportion of meniscus tears considered repairable, and thus repaired, increased during a single surgeon’s practice of 20 years. Arthrosc Sports Med Rehabil 5(5):100778. 10.1016/j.asmr.2023.100778 37560143 10.1016/j.asmr.2023.100778 PMC10407626

[4] Chan EW, Chaulk RC, Cheng Y, Shin J (2021) No decrease in incidence of arthroscopic meniscectomy in a Canadian province. Knee Surg Sports Traumatol Arthrosc 29(12):4223–4231. 10.1007/s00167-021-06534-7 33745007 10.1007/s00167-021-06534-7

[5] Migliorini F, Schäfer L, Bell A, Weber CD, Vecchio G, Maffulli N (2023) Meniscectomy is associated with a higher rate of osteoarthritis compared to meniscal repair following acute tears: a meta-analysis. Knee Surg Sports Traumatol Arthrosc. 10.1007/s00167-023-07600-y 37812251 10.1007/s00167-023-07600-y PMC10719156

[6] Elattar M, Dhollander A, Verdonk R, Almqvist KF, Verdonk P (2011) Twenty-six years of meniscal allograft transplantation: is it still experimental? A meta-analysis of 44 trials. Knee Surg Sports Traumatol Arthrosc 19(2):147–157. 10.1007/s00167-010-1351-6 21161170 10.1007/s00167-010-1351-6

[7] Getgood A, LaPrade RF, Verdonk P, Gersoff W, Cole B, Spalding T, Group I (2017) International Meniscus Reconstruction Experts Forum (IMREF) 2015 consensus statement on the practice of meniscal allograft transplantation. Am J Sports Med 45(5):1195–1205. 10.1177/0363546516660064 27562342 10.1177/0363546516660064

[8] Leite CBG, Merkely G, Zgoda M, Farina EM, Görtz S, Howard J, Lattermann C (2023) Systematic review of clinical results after medial meniscus allograft transplantation reveals improved patient reported outcomes at greater than 5 years follow-up. Arthroscopy 39(3):802–811. 10.1016/j.arthro.2022.11.033 36543661 10.1016/j.arthro.2022.11.033

[9] Novaretti JV, Patel NK, Lian J, Vaswani R, de Sa D, Getgood A, Musahl V (2019) Long-term survival analysis and outcomes of meniscal allograft transplantation with minimum 10-year follow-up: a systematic review. Arthroscopy 35(2):659–667. 10.1016/j.arthro.2018.08.031 30712641 10.1016/j.arthro.2018.08.031

[10] Colombini A, Libonati F, Lopa S, Peretti GM, Moretti M, de Girolamo L (2022) Autologous chondrocyte implantation provides good long-term clinical results in the treatment of knee osteoarthritis: a systematic review. Knee Surg Sports Traumatol Arthrosc. 10.1007/s00167-022-07030-2 35716187 10.1007/s00167-022-07030-2

[11] Colombini A, Raffo V, Gianola S, Castellini G, Filardo G, Lopa S et al (2024) Matrix-assisted autologous chondrocyte transplantation is effective at mid/long-term for knee lesions: a systematic review and meta-analysis. Knee Surg Sports Traumatol Arthrosc. 10.1002/ksa.12549 39624924 10.1002/ksa.12549 PMC12310092

[12] Wang AS, Nagelli CV, Lamba A, Saris DBF, Krych AJ, Hevesi M (2024) Minimum 10-year outcomes of matrix-induced autologous chondrocyte implantation in the knee: a systematic review. Am J Sports Med 52(9):2407–2414. 10.1177/03635465231205309 38312085 10.1177/03635465231205309 PMC12456864

[13] Harris JD, Cavo M, Brophy R, Siston R, Flanigan D (2011) Biological knee reconstruction: a systematic review of combined meniscal allograft transplantation and cartilage repair or restoration. Arthroscopy 27(3):409–418. 10.1016/j.arthro.2010.08.007 21030203 10.1016/j.arthro.2010.08.007

[14] Kempshall PJ, Parkinson B, Thomas M, Robb C, Standell H, Getgood A, Spalding T (2015) Outcome of meniscal allograft transplantation related to articular cartilage status: advanced chondral damage should not be a contraindication. Knee Surg Sports Traumatol Arthrosc 23(1):280–289. 10.1007/s00167-014-3431-5 25432522 10.1007/s00167-014-3431-5

[15] Lee BS, Kim HJ, Lee CR, Bin SI, Lee DH, Kim NJ, Kim CW (2018) Clinical outcomes of meniscal allograft transplantation with or without other procedures: a systematic review and meta-analysis. Am J Sports Med 46(12):3047–3056. 10.1177/0363546517726963 28945482 10.1177/0363546517726963

[16] Shamseer L, Moher D, Clarke M, Ghersi D, Liberati A, Petticrew M et al. (2015) Preferred reporting items for systematic review and meta-analysis protocols (PRISMA-P) 2015: elaboration and explanation. BMJ. 10.1136/bmj.g7647 25555855 10.1136/bmj.g7647

[17] Gilat R, Cole BJ (2020) Meniscal allograft transplantation: indications, techniques, outcomes. Arthroscopy 36(4):938–939. 10.1016/j.arthro.2020.01.025 32006568 10.1016/j.arthro.2020.01.025

[18] Crawford K, Briggs KK, Rodkey WG, Steadman JR (2007) Reliability, validity, and responsiveness of the IKDC score for meniscus injuries of the knee. Arthroscopy 23(8):839–844. 10.1016/j.arthro.2007.02.005 17681205 10.1016/j.arthro.2007.02.005

[19] Hambly K, Griva K (2008) IKDC or KOOS? Which measures symptoms and disabilities most important to postoperative articular cartilage repair patients? Am J Sports Med 36(9):1695–1704. 10.1177/0363546508317718 18577582 10.1177/0363546508317718

[20] Slim K, Nini E, Forestier D, Kwiatkowski F, Panis Y, Chipponi J (2003) Methodological index for non-randomized studies (minors): development and validation of a new instrument. ANZ J Surg 73(9):712–716. 10.1046/j.1445-2197.2003.02748.x 12956787 10.1046/j.1445-2197.2003.02748.x

[21] Álvarez-Lozano E, Luna-Pizarro D, Meraz-Lares G, Quintanilla-Loredo R, Cerdá-García MV, Forriol F (2022) Two-stage bone and meniscus allograft and autologous chondrocytes implant for unicompartmental osteoarthritis: midterm results. Musculoskelet Surg 106(2):133–143. 10.1007/s12306-020-00680-w 32845424 10.1007/s12306-020-00680-w

[22] Yoon KH, Kang SG, Kwon YB, Kim EJ, Kim SG (2019) Clinical outcomes and survival rate of autologous chondrocyte implantation with and without concomitant meniscus allograft transplantation: 10- to 15-year follow-up study. Arch Orthop Trauma Surg 139(8):1117–1123. 10.1007/s00402-019-03148-0 30830306 10.1007/s00402-019-03148-0

[23] Ogura T, Bryant T, Minas T (2016) Biological knee reconstruction with concomitant autologous chondrocyte implantation and meniscal allograft transplantation: mid- to long-term outcomes. Orthop J Sports Med 4(10):2325967116668490. 10.1177/2325967116668490 27803938 10.1177/2325967116668490 PMC5076751

[24] Bhosale AM, Myint P, Roberts S, Menage J, Harrison P, Ashton B et al. (2007) Combined autologous chondrocyte implantation and allogenic meniscus transplantation: a biological knee replacement. Knee 14(5):361–368. 10.1016/j.knee.2007.07.002 17689085 10.1016/j.knee.2007.07.002

[25] Farr J, Rawal A, Marberry KM (2007) Concomitant meniscal allograft transplantation and autologous chondrocyte implantation: minimum 2-year follow-up. Am J Sports Med 35(9):1459–1466. 10.1177/0363546507301257 17435058 10.1177/0363546507301257

[26] Cvetanovich GL, Riboh JC, Tilton AK, Cole BJ (2017) Autologous chondrocyte implantation improves knee-specific functional outcomes and health-related quality of life in adolescent patients. Am J Sports Med 45(1):70–76. 10.1177/0363546516663711 27566240 10.1177/0363546516663711

[27] Rue JP, Yanke AB, Busam ML, McNickle AG, Cole BJ (2008) Prospective evaluation of concurrent meniscus transplantation and articular cartilage repair: minimum 2-year follow-up. Am J Sports Med 36(9):1770–1778. 10.1177/0363546508317122 18483199 10.1177/0363546508317122

[28] Pawelczyk J, Fanourgiakis I, Feil S, Siebold R (2024) Significant improvements in clinical outcome measures and patient satisfaction after combined all-arthroscopic meniscal allograft transplantation and autologous chondrocyte implantation: a single-centre longitudinal study. Knee Surg Sports Traumatol Arthrosc 32(1):78–88. 10.1002/ksa.12023 38226734 10.1002/ksa.12023

[29] Gersoff WK (2002) Combined meniscal allograft transplantation and autologous chondrocyte implantation. Oper Tech Sports Med 10(3):165–167. 10.1053/otsm.2002.36440

[30] Liu JN, Gowd AK, Redondo ML, Christian DR, Cabarcas BC, Yanke AB, Cole BJ (2019) Establishing clinically significant outcomes after meniscal allograft transplantation. Orthop J Sports Med 7(1):2325967118818462. 10.1177/2325967118818462 30643837 10.1177/2325967118818462 PMC6322105

[31] Farr J, Lewis P, Cole BJ (2004) Patient evaluation and surgical decision making. J Knee Surg 17(4):219–228. 10.1055/s-0030-1248226 15553590 10.1055/s-0030-1248226

[32] Obremskey WT, Pappas N, Attallah-Wasif E, Tornetta P 3rd, Bhandari M (2005) Level of evidence in orthopaedic journals. J Bone Joint Surg Am 87(12):2632–2638. 10.2106/jbjs.E.00370 16322612 10.2106/JBJS.E.00370

