# Supplementary material for: Clinical results and failure rates after meniscal allograft transplantation and autologous chondrocyte implantation: a systematic review
Source: Knee Surg Relat Res. 2025 Sep 22;37:39. doi: 10.1186/s43019-025-00291-4 (PMC12455755; doi:10.1186/s43019-025-00291-4)
Supplement: Supplementary file 1 — Supplementary material 1. Table 1 Exact search string used for the comprehensive literature search in each database. [file 43019_2025_291_MOESM1_ESM.docx]

**Supplementary Table 1. Search Strategy.**

| **Search Strategy** |
| --- |
| *Medline via PubMed and PubMed Central* |
| ((((((meniscal allograft transplantation) OR (meniscus allograft transplantation)) OR (meniscal allograft)) OR (meniscus allograft)) OR (meniscal transplantation)) OR (meniscus transplantation)) AND ((autologous chondrocyte implantation) OR (autologous chondrocyte transplantation)) |
| *Cochrane Library* |
| ((((((meniscal allograft transplantation) OR (meniscus allograft transplantation)) OR (meniscal allograft)) OR (meniscus allograft)) OR (meniscal transplantation)) OR (meniscus transplantation)) AND ((autologous chondrocyte implantation) OR (autologous chondrocyte transplantation)) |
| *Web of Science Core Collection* |
| (((ALL=(autologous chondrocyte implantation))) OR ALL=(autologous chondrocyte transplantation)) AND ((((((ALL=(meniscal allograft transplantation)) OR ALL=(meniscus allograft transplantation)) OR ALL=(meniscal transplantation)) OR ALL=(meniscus transplantation) OR ALL=(meniscal allograft)) OR ALL=(meniscus allograft))) |

Exact search string used for the comprehensive literature search in each database.
